# Clinical Manifestation and Molecular Characterization of a Novel Member of the *Nannizziopsiaceae* in a Pulmonary Granuloma From a Galapagos Tortoise (*Chelonoidis nigra*)

**DOI:** 10.3389/fvets.2020.00024

**Published:** 2020-02-07

**Authors:** Jane E. Christman, Amy B. Alexander, Kyle A. Donnelly, Robert J. Ossiboff, Nicole I. Stacy, Rebecca L. Richardson, J. Brad Case, April L. Childress, James F. X. Wellehan

**Affiliations:** ^1^Department of Comparative, Diagnostic and Population Medicine, College of Veterinary Medicine, University of Florida, Gainesville, FL, United States; ^2^Department of Small Animal Clinical Sciences, College of Veterinary Medicine, University of Florida, Gainesville, FL, United States

**Keywords:** onygenales, *Nannizziopsiaceae*, fungal pneumonia, *Chelonoidis nigra*, Galapagos tortoise

## Abstract

Nannizziopsiaceae is a family of fungal organisms within the order Onygenales containing two genera of important reptile pathogens, *Nannizziopsis* and *Paranannizziopsis*. A captive Galapagos tortoise (*Chelonoidis nigra*) from Boca Raton, Florida, United States, was presented for a clinical history of chronic progressive lethargy and inappetence. At initial presentation, the tortoise had a moderate non-regenerative anemia, leukocytosis, whip-like heterophil projections, erythrocyte fragmentation, and fibrin strands, with the latter two raising concern for disseminated intravascular coagulation. A single large encapsulated pulmonary granuloma was identified through imaging, including plain film radiography and bronchoscopy. Direct intralesional samples were obtained from transcarapacial celioscopy for fungal culture, cytology, histopathology, and polymerase chain reaction. Amplification and sequencing of the ITS2 region of the rRNA genes with Bayesian and maximum likelihood analyses placed the fungus in the family Nannizziopsiaceae within the order Onygenales, representing a novel fungal species.

## Introduction

Galapagos tortoises (*Cheloinidis nigra*) are the largest tortoise species and are listed in CITES Appendix I. They appear prone to fungal infections in captivity, with reported organisms including *Beauveria bassiana* (1)*, Exophiala equina* (2), and *Aphanoascella galapagosensi* (3).

The fungal order Onygenales in the class Eurotiomycetes contains the majority of the most significant fungal pathogens of vertebrates, including *Ajellomyces* (*Blastomyces* and *Histoplasma*), *Coccidioides, Paracoccidioides, Microsporum, Trichophyton, Ophidiomyces*, and *Lacazia*. Within the order Onygenales, members of the family Nannizziopsiaceae contain the genera *Nannizziopsis* and *Paranannizziopsis*, which cause significant morbidity and mortality in reptiles (4). While many cases were initially misidentified as *N. vriesii* based on morphology, and the initial isolation of *Nannizziopsis* (*Rollandina*) *vriesii* was from an *Amieva* sp., the authors are not aware of any reptile isolates other than the one in 1970, and it does not appear to be a common reptile pathogen. *N*. *guarroi* is the most widespread in captive reptiles, and most significantly impacts squamates in Iguania (Agamidae, Chamaeleonidae, Iguanidae, and relatives), where it is commonly known as “yellow fungus disease” (5). *N. arthrosporioides, N. barbata, N. chlamydospora*, and *N. draconii* reportedly cause cutaneous infections in agamids, albeit less commonly, while *N. dermatitidis* displays a similar clinical presentation in chameleons and leopard geckos. *N. crocodili* has been associated with disease in crocodiles and is the only member of the genus primarily known to cause infections in non-squamate reptiles (6). *N. obscura* has been linked to osteomyelitis in humans (7) and a *N. obscura*-like fungus has been found to cause fungal disease in a Bryde's whale (*Balaenoptera edeni*) (8). The genus *Paranannizziopsis* causes disease primarily in aquatic snakes, although disease has also been seen in tuatara and a coastal bearded dragon (9). To date, disease due to Nannizziopsiaceae has not been described in the Testudines.

## Case Presentation

A captive, ~80-year-old male Galapagos tortoise (*Chelonoidis nigra*) from Boca Raton, Florida, USA, presented for 8 months of progressive lethargy and a 2-week duration of inappetence. The tortoise was originally wild caught from the Galapagos Islands in the 1940s and had been maintained in the current collection for over 30 years while housed in close proximity to several other chelonian species in intermixed groups. Prior to presentation, the animal was treated with three courses of unknown doses of fenbendazole separated 3 months apart.

**Figure 1:**
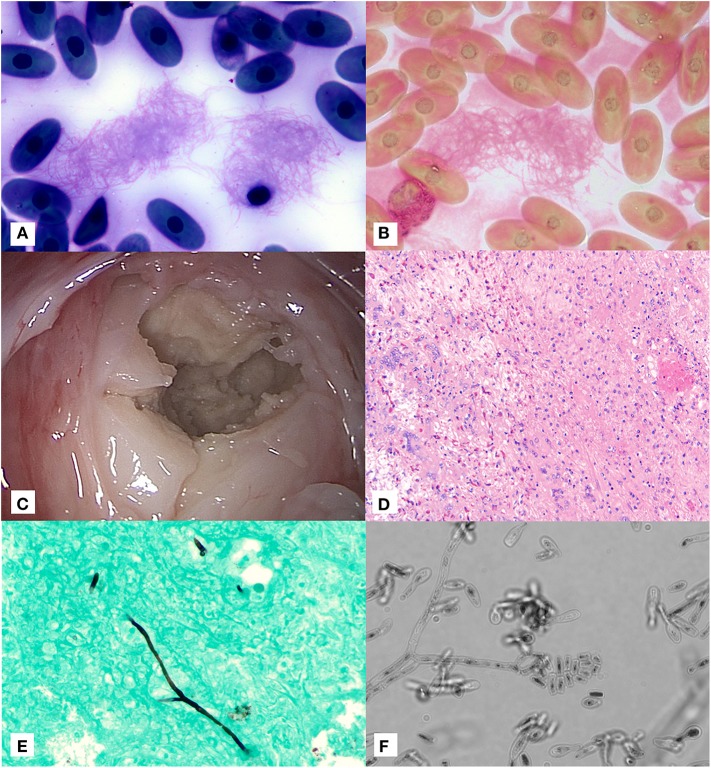
Image composite of diagnostic findings in a Galapagos tortoise (*Chelonoidis nigra*) with a pulmonary fungal granuloma caused by a novel Nannizziopsiaceae. **(A)** Aggregates of fine linear fibrin strands in a blood film at time of initial presentation stained with Wright-Giemsa (×100 objective). **(B)** Fraser-Lendrum stain of fibrin strands (×100 objective). **(C)** Gross image of encapsulated pulmonary mass through transcarapacial pulmonoscopy. **(D)** Hematoxyin/Eosin staining of pulmonary granuloma. **(E)** Gomori methenamine silver staining of rare branching, parallel walled fungal hyphae within the pulmonary granuloma as sampled by direct intralesional sampling (×20 objective). **(F)** Direct light microscopy of fungal microconidia and hyphae as grown in culture (×60 objective).

On presentation, the tortoise was severely lethargic and unable to lift his head. He was subjectively considered in adequate body condition based on musculature. A moderate amount of opaque tan mucoid discharge was noted on the nasal planum and oral cavity. A corneal opacity was also present in the left eye, and ophthalmic examination noted intracorneal hemorrhage and corneal edema. A complete blood count at initial presentation revealed a mild to moderate leukocytosis (18.2K cells/μl; expected reference 0.5–15.9 × 10^3^ cells/μl (10) with a mild heterophil left shift (0.9 × 10^3^ cells/μl) as well as severe, non-regenerative anemia [PCV 14%; expected reference 8-29.8%; (10)] with occasional erythrocyte fragments. Initial blood film review resulted in identification of variably sized aggregates of fine linear strands suggestive of fibrin with entrapped thrombocytes (Figure 1A); these linear strands were confirmed as fibrin based on Fraser Lendrum stain (Figure 1B). The initial and subsequent blood films showed variable numbers of whip-like heterophil projections suggestive of inflammation (11). Together, the presence of fibrin strands and evidence of erythrocyte fragmentation raised concern for developing disseminated intravascular coagulation. Plasma chemistry results did not show any abnormal findings.

Transcarapacial radiographs pursued 48 h after admission showed an expansile soft tissue opacity in the right dorsal lung with a mild to moderate faveolar to interstitial pattern in the left lung. Blood culture performed at the time of ultrasound grew aerobic, fastidious, Gram-positive bacilli that could not be identified biochemically. Sensitivity could not be performed due to fastidious growth. On bronchoscopy, just past the branch to the right bronchi, a large, tan mass was identified. Superficial biopsy samples were submitted for cytology, histopathology, fungal culture, and fungal polymerase chain reaction (PCR). Fungal culture was negative. Cytology showed abundant mucus with mixed heterophilic and histiocytic inflammation with focal aggregates of coccobacilli, and a single fragment of fungal hypha stained argyrophilic with Gomori methenamine stain. Histologically the biopsy consisted of a ciliated respiratory epithelium overlying a submucosa mildly expanded with fibrillar to homogenous eosinophilic material and multifocal aggregates of lymphocytes, plasma cells, macrophages, and heterophils; no fungal elements were identified, and no microbes were identified on Gram, Gomori methenamine-silver (GMS), periodic acid-Schiff, or Fite's acid fast stained slides.

Five days after initial presentation, transcarapacial surgical pulmonoscopy was performed. Two adjacent 5 mm pilot holes were created in the right cranial carapace at the second pleural scute. A large (~10 cm in diameter) round tan granuloma was observed along the dorsal pulmonary parenchyma within the right lung (Figure 1C). The capsule was debrided and deep tissue biopsies were collected. Voriconazole (0.2 mg/kg, VFend, Pfizer, New York, NY) and amphotericin B (0.1 mg/kg, X-Gen Pharmaceuticals Inc., Horseheads, NY) were injected directly into the center of the granuloma. A 12-french, 11 cm polypropylene intralesional port (Boston Scientific, Marlborough, MA) was placed directly within the granuloma. Histology showed granulomatous and heterophilic inflammation with mixed proteinaceous cellular debris, reactive faveolar pneumocytes, and low numbers of fungal hyphae (Figure 1D). GMS staining highlighted fungal hyphae with septate parallel walls 4-6 μm in diameter and infrequent dichotomous acute angle branching (Figure 1E). No acid fast organisms were seen on Fite's acid fast staining.

The lung granuloma sample was plated onto Chocolate agar, Columbia Blood agar (BAP), MacConkey agar (MAC), Colistin with Nalidixic acid (CNA), Potato Flake agar (PFA), and Inhibitory Mold agar (IMA). The Chocolate, BAP, MAC, and CNA plates were incubated at 25 ± 2°C for 5 days. Plates were reviewed every 18-24 h for bacterial growth. On day 5, a very scant amount of filamentous growth appeared around the tissue that was embedded into the Chocolate, BAP and CNA agar plates. No bacterial colonies appeared after 5 days. The fungal colony was isolated to a PFA and incubated at 25 ± 2°C, along with the original PFA plates that were set-up for the fungal culture, to ensure growth from the aerobic culture matched what was growing on the fungal culture.

The PFA and IMA plates were incubated at 25 ± 2° and reviewed at day 7. Very scant growth of a white, dense fungus appeared on each plate. Microscopic morphology was reviewed using the cellophane tape mount method. Hyphae were septate and produced arthroconidia-like structures. Conidia were tear-drop shaped with blunt or straight bases. Some were shaped like glass bottles, with a spherical base and long, straight neck. Most conidia were single celled, and some had 2 cells (Figure 1F).

DNA was extracted from a biopsy of the pulmonary granuloma and from a fungal culture with a commercial extraction kit (DNeasy® Blood and Tissue Kit, Qiagen, Valencia, CA) following manufacturer's instructions. Polymerase chain reaction amplification of the internal transcribed spacer 2 (ITS2) domain of the rRNA gene was undertaken using previously described methods with primers ITS4 and ITS86 (12) on both the direct sample taken directly from the celioscopy as well as from fungal colonies taken from the fungal culture. To obtain additional sequence, polymerase chain reaction amplification of the actin gene using primers Act-l b and Act-4r was done on the samples (13). Products were electrophoresed on a 1% agarose gel and PCR products were purified using a commercial extraction kit (QIAquick gel extraction kit, Qiagen Inc., Valencia, CA) followed by direct sequencing using a commercial kit (Big-Dye terminator kit, Applied Biosystems, Foster City, CA) on an automated DNA sequencer (ABI 3130, Applied Biosystems, Foster City, CA). Primer sequences were trimmed off prior to further analysis, and the ITS2 sequence was 248 nucleotides and the actin sequence was 852 nucleotides. Sequences were submitted to GenBank under accession numbers MN403040 and (submitted, to be added prior to publication).

**Figure 2:**
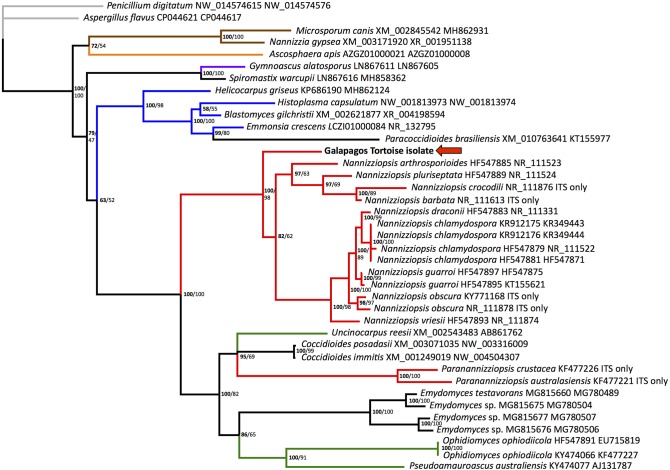
Bayesian tree depicting the relationship of the Galapagos tortoise fungus to other members of the order Onygenales, based on the ITS2 region. Numbers at each node represent the posterior probability in bold, and ML bootstrap values are given unbolded to the right. *Penicillium digitatum* is used as an outgroup, and the order Eurotiales is in gray. The family Arthrodermataceae is in brown, Ascosphaeraceae is in orange, Gymnoascaceae is in purple, Ajellomycetaceae is in blue, Nannizziopsiaceae are in red, Onygenaceae are in green, and taxa not currently assigned to a family are in black. The Galapagos tortoise fungus is marked with an arrow.

Homologous nucleotide sequences for fungal ITS2 and actin were retrieved from GenBank and aligned using the program Multiple Alignment using Fast Fourier Transform (MAFFT) (14). All *Paranannizziopsis* and several *Nannizziopsis* species had no available actin sequences. *Penicillium_digitatum* (GenBank accession numbers NW_014574615 and NW_014574576), a fungus in the order Eurotiales in the class Eurotiomycetes, was designated as the outgroup. Bayesian phylogenetic analyses of nucleotide alignments were performed using MrBayes 3.1.2 on the CIPRES server, with a general time reversible model, gamma distributed rate variation, and a proportion of invariant sites (15, 16). The first 25% of 2,000,000 iterations were discarded as burn in. There were no significant topological differences when each gene was run individually, so the genes were concatenated, with ambiguities used for actin in the 2 *Paranannizziopsis* and 4 *Nannizziopsis* that had no available sequence. The Bayesian phylogenetic tree is shown (Figure 2).

Maximum likelihood analysis was performed using RAxML on the CIPRES server, using a gamma distributed rate variation, a proportion of invariant sites, and a general time reversible model (17). Again, *Penicillium digitatum* (GenBank accession numbers NW_014574615 and NW_014574576) was used as an outgroup. To test the strength of the tree topology, bootstrap analysis was used (1000 re-samplings) (18). ML bootstrap values are shown on the Bayesian tree (Figure 2).

Samples were submitted to the University of Texas San Antonio for fungal susceptibility, which showed the organism was inhibited by itraconazole at 0.06 μg/ml, posaconazole at <0.03 μg/ml, and terbinafine at <0.004 μg/ml. While there are no CLSI standards, these values were interpreted by the authors as likely to be clinically efficacious. Inhibition by amphotericin B occurred at 0.25 μg/ml, by fluconazole at 32 μg/ml, and by voriconazole at 0.125 μg/ml. The patient was discharged on itraconazole (6.5 mg/kg q24h, Par Pharmacueticals, Chestnut Ridge, NY) and terbinafine (3.8 mg/kg q24h, Harris Pharmaceutical Inc., Fort Myers, FL). While follow-up imaging was declined, the moribund state of the state of the tortoise resolved and spontaneous hemorrhage was no longer seen. The patient is known to be doing well 2 years after discharge.

## Discussion

Fungi in the order Onygenales (class Eurotiomycetes) show significant genomic adaptation to utilizing animal hosts, with reduction in cellulose binding genes for digesting plants and an increase in keratinase genes for digesting animals (19, 20). Despite the clinical significance of the Onygenales in diverse vertebrate hosts, there is little data on disease due to the Onygenales in Testudines to date. *Aphanoascella galapagoensis*, a member of the Onygenales in the family Onygenaceae, was isolated from a Galapagos tortoise with carapacial keratitis (3). Recently, *Emydomyces testavorans*, a member of the Onygenales that does not cluster with the Nannizziopsiaceae, was reported in shell lesions from diverse aquatic turtles (21).

The ITS2 region is transcribed with the highly conserved rRNA genes but does not have an apparent function and is therefore not highly conserved. This results in utility for distinguishing closely related organisms but presents challenges with longer-range phylogenetic analyses. Actin is an important structural gene and is more conserved. Support for some family-level relationships was fairly strong, with Arthrodermataceae and Ajellomycetaceae (shown in Figure 2 in brown and blue, respectively) showing 100% posterior probability and 98-100% ML bootstrap values. However, the monophyly of *Nannizziopsis* and *Paranannizziopsis* was not identified using this region. Our analysis found moderate support for *Paranannizziopsis* clustering within Onygenaceae, with 100% posterior probability and 82% ML bootstrap support. It should be noted that we did not have actin sequence for *Paranannizziopsis*, so this placement is based solely on ITS2 data. Even with additional gene sequences, strong support for this was not found in some other analyses (21). Use of additional genes would resolve placement of *Paranannizziopsis*. However, the support for clustering of the Galapagos tortoise isolate with *Nannizziopsis* is strong; the Bayesian posterior probability is 100% and the ML bootstrap value is 98%. This organism should be classified either in *Nannizziopsis* or as a novel sister genus. The genetic distances seen within the genus in our analysis are greater than distances between some other genera, suggesting a novel sister genus may be more appropriate.

While reported *Nannizziopsis* cases in squamates and crocodilians presented initially as cutaneous lesions, pulmonary disease has been reported in an *Amieva* sp. (22) and a carpet chameleon (*Furcifer* [*Chamaeleo*] *lateralis*) (23). *Nannizziopsis hominis* has been isolated from fungal lesions in the lungs of humans (4). The findings in this tortoise were most consistent with a primary pulmonary infection, as no cutaneous lesions were identified.

Giant tortoises represent a diagnostic challenge. The shell of tortoises is a significant impediment to interpretation of traditional radiographic imaging, and advanced imaging such as computed tomography (CT) or magnetic resonance imaging (MRI) is often needed to image soft tissue structures (24, 25). As are many giant tortoises, this patient was too large to fit in available CT or MRI equipment. Endoscopy represents an alternative methodology for visualization of lesions in tortoises (26). Pulmonary fungal disease is not uncommon in Galapagos tortoises (1, 27). Transcarapacial pulmonoscopy has been used for fungal pneumonia in a smaller tortoise species (28); without its use in this case, a definitive diagnosis could not have been made and direct treatment of the lesion would not have been possible.

Other factors may have contributed to the development of clinical disease in this patient. Benzimidazole toxicosis affects rapidly dividing cells, especially those of hematopoietic tissue; Hermann's tortoises (*Testudo hermanni*) given two 5-day courses of 50 mg/kg of fenbendazole 2 weeks apart showed significant heteropenia for 70 days (29). The fenbendazole treatment prior to admission may have predisposed this tortoise to immunosuppression and fungal infection. The climate in Boca Raton, FL, may also have played an immunosuppressive role. Puerto Ayora, in the Galapagos Islands, has average temperatures of 26.4°C during the hottest month of the year, and 21.4°C during the coolest month of the year (https://en.climate-data.org). Palm Beach, 45 km north of Boca Raton, has average temperatures of 28°C during the hottest month of the year, and 18.6°C during the coolest month of the year (https://en.climate-data.org). Temperatures outside the range for which ectotherms are adapted to can be expected to result in suboptimal immune function (30, 31).

The non-regenerative anemia on initial bloodwork reflects the patient's debilitated status and suggested bone marrow suppression secondary to chronic inflammation and/or from presumptive drug toxicity. The observation of fibrin strands on blood films at initial presentation was an unusual finding and raised concern for developing intravascular coagulation, although thrombocytes were considered adequate and observed in variably sized clumps, some of which were entrapped in fibrin aggregates. This may also explain the presence of the intracorneal hemorrhage and subjectively prolonged bleeding at the site of collection during venipuncture at admission. Disseminated intravascular coagulation has been previously reported in other chelonians, including a softshell turtle and sea turtles in response to mycobacteriosis (32) and cold stunning (33), respectively. Significant improvement was noted during the 4 weeks of hospitalization at the clinic. The patient was discharged and has been clinically stable at time of writing of this manuscript (18 months after admission).

The relatively low concentration at which itraconazole inhibited this fungus *in vitro* may have been important for the response to therapy. This does not appear to be the case for all members of the *Nannizziopsiaceae*. In one report, two of three bearded dragons treated for *N. guarroi* with itraconazole died (34). In another report, six of seven bearded dragons treated for *N. guarroi* with itraconazole and clotrimazole died (35) Only one of two chameleons treated for *N. dermatitidis* with itraconazole survived (23). Phenotypic differences between species in the *Nannizziopsiaceae* may be clinically significant, emphasizing the importance of accurate and precise fungal identification for patient care. This case report describes the clinical manifestation and medical management of Nanniziomycotic infection in a tortoise and documents the identification and molecular characterization of a novel fungal pathogen in a clinically significant clade.

## Data Availability Statement

The sequence data from this study can be found in GenBank under accession number MN403040.

## Author Contributions

Clinical management of the case by JEC, AA, KD, JW. Transcarapacial pulmonoscopy, sample collection, and port placement by JBC, KD. Fungal culture and morphologic characterization by RR. Histologic interpretation performed by RO. Cytologic interpretation performed by NS. PCR and sequencing performed by AC. Sequence interpretation and phylogenetic analysis by JW. Initial draft written by JEC. All authors contributed to the writing and editing of this manuscript.

### Conflict of Interest

The authors declare that the research was conducted in the absence of any commercial or financial relationships that could be construed as a potential conflict of interest.
